# Supplementary material for: The causal role of circulating inflammatory markers in osteoporosis: a bidirectional Mendelian randomized study
Source: Front Immunol. 2024 Jul 18;15:1412298. doi: 10.3389/fimmu.2024.1412298 (PMC11291241; doi:10.3389/fimmu.2024.1412298)
Supplement: Supplementary file 1 [file DataSheet_1.zip › supplementary material/OSTEOPOROSIS-CIM/GCST90274825.tsv.gz_leave_one_out.pdf]

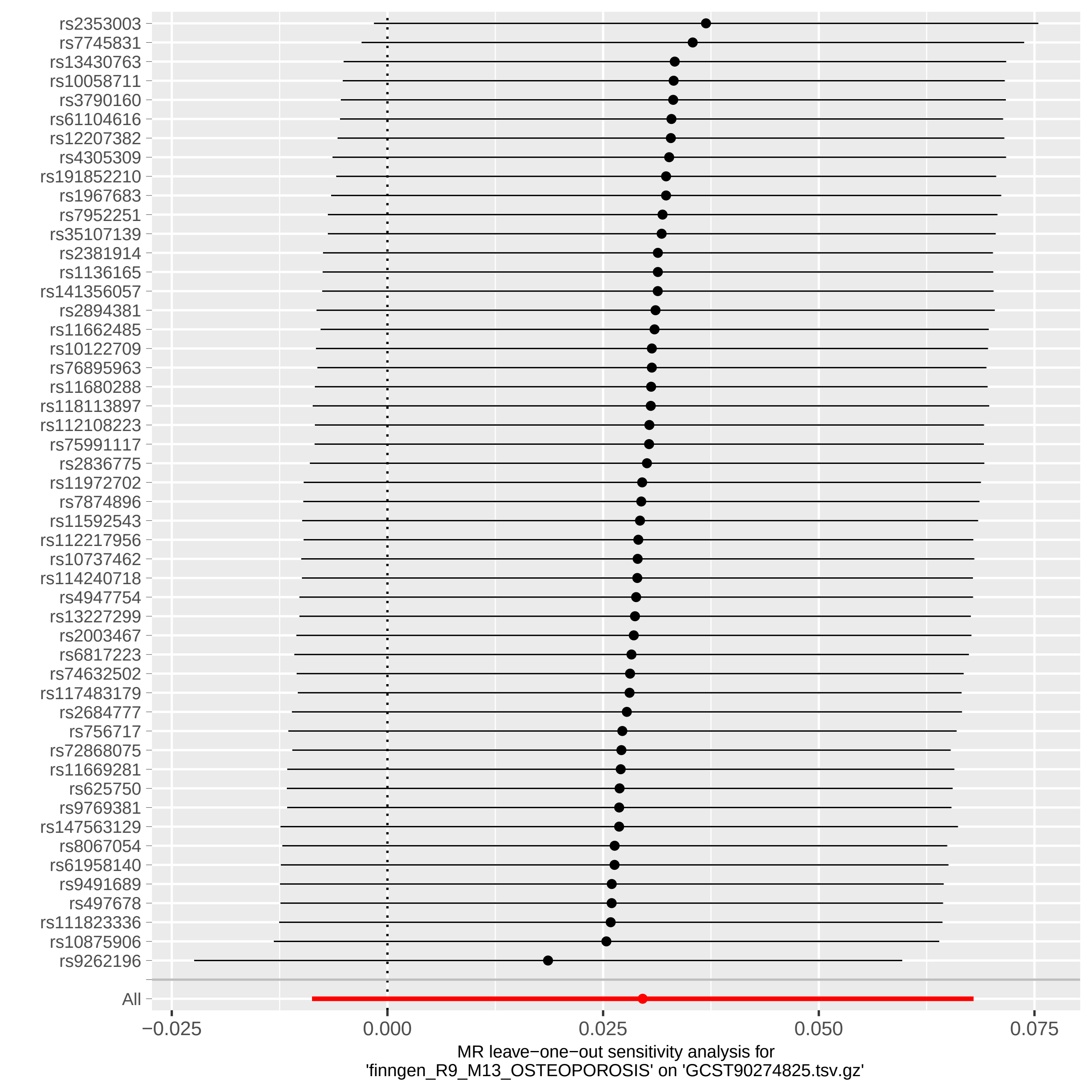

rs2353003
rs7745831
rs13430763
rs10058711
rs3790160
rs61104616
rs12207382
rs4305309
rs191852210
rs1967683
rs7952251
rs35107139
rs2381914
rs1136165
rs141356057
rs2894381
rs11662485
rs10122709
rs76895963
rs11680288
rs118113897
rs112108223
rs75991117
rs2836775
rs11972702
rs7874896
rs11592543
rs112217956
rs10737462
rs114240718
rs4947754
rs13227299
rs2003467
rs6817223
rs74632502
rs117483179
rs2684777
rs756717
rs72868075
rs11669281
rs625750
rs9769381
rs147563129
rs8067054
rs61958140
rs9491689
rs497678
rs111823336
rs10875906
rs9262196
All
−0.025
0.000
0.025
0.050
0.075
MR leave−one−out sensitivity analysis for
'finngen_R9_M13_OSTEOPOROSIS' on 'GCST90274825.tsv.gz'
